# Consensus Guidelines and Recommendations for The CD38 Monoclonal Antibody-based Quadruplet Therapy and Management in Clinical Practice for Newly Diagnosed Multiple Myeloma: From the Pan-Pacific Multiple Myeloma Working Group

**DOI:** 10.46989/001c.133682

**Published:** 2025-04-11

**Authors:** Wenming Chen, Zhen Cai, James CS Chim, Wee Joo Chng, Juan Du, Chengcheng Fu, Ichiro Hanamura, Jian Hou, Jeffrey Shang-Yi Huang, Tadao Ishida, Aijun Liu, Vadim Ptushkin, Anastasiya Semenova, Naoki Takezako, Raymond Siu Ming Wong

**Affiliations:** 1 Department of Hematology, Myeloma Research Center of Beijing Beijing Chaoyang Hospital, Capital Medical University, Beijing, China; 2 School of Medicine First Affiliated Hospital Zhejiang University, China; 3 University of Hong Kong, Hong Kong; 4 National University Cancer Institute, Singapore; 5 Myeloma & Lymphoma Center Shanghai Changzheng Hospital, China; 6 First Affiliated Hospital of Soochow University, China; 7 Division of Hematology, Department of Internal Medicine Aichi Medical University, Japan; 8 Department of Hematology Renji Hospital, School of Medicine, Shanghai Jiao Tong University, China; 9 National Taiwan University Hospital, Taiwan; 10 Japanese Red Cross Medical Center, Japan; 11 Botkin Moscow City Clinical Hospital, Russia; 12 Russian Cancer Research Center named after N.N. Blokhin RAMS, Russia; 13 Nerima Hikarigaoka Hospital, Japan; 14 Sir Y.K. Pao Centre for Cancer & Department of Medicine and Therapeutics Prince of Wales Hospital, The Chinese University of Hong Kong, Hong Kong SAR

**Keywords:** Multiple myeloma, Anti-CD38 monoclonal antibody, Newly diagnosed, Quadruplet therapy, Isatuximab, Daratumumab

## Abstract

The therapeutic outcomes of clinical trials for incorporating anti-CD38 monoclonal antibodies (including isatuximab and daratumumab) into the bortezomib/lenalidomide/dexamethasone (VRd) triplet therapy backbone as the first-line treatment for newly diagnosed multiple myeloma (NDMM) have demonstrated significant improved efficacies. From a safety perspective, the addition of anti-CD38 monoclonal antibodies into the triplet therapies did not raise additional safety concerns. Based on the promising results, the National Comprehensive Cancer Network (NCCN) Guidelines Version 1.2025 had updated the quadruplet therapy incorporating anti-CD38 monoclonal antibodies with VRd-based therapies as the primary therapy for both transplantation-eligible and transplantation-ineligible NDMM patients. Thus, a panel of experts in hematology and oncology with extensive experience in the treatment of NDMM was convened in 2024 to develop consensus recommendations based on recent evidence from pivotal clinical trials and real-world practices, providing clear guidance for optimizing treatment strategies in both transplantation-eligible and transplantation-ineligible patients. The main topics identified for discussion and recommendation were: (i) the benefits and indications for quadruplet therapy for NDMM; (ii) the optimization of quadruplet therapy strategies; (iii) the management and monitoring of potential adverse events for quadruplet therapy, and (iv) the impact of quadruplet regimens on tandem stem cell transplantation and maintenance treatment. Recommendations were then presented to the entire panel for further discussion and amendment before voting. This manuscript presents the recommendations developed, including ﬁndings from the expert panel discussions, consensus recommendations and a summary of evidence supporting each recommendation.

## Introduction

The treatment of newly diagnosed multiple myeloma (NDMM) has undergone significant advancements in recent years, driven by the development of novel therapeutic regimens that offer deeper and more durable responses. Among these, the VRd regimen (bortezomib, lenalidomide, and dexamethasone) has emerged as a cornerstone in both transplantation-eligible and transplantation-ineligible patient populations. Over the past decade, triplet therapy regimens have firmly established their therapeutic efficacy, with adverse events (AEs) that are relatively predictable in clinical practice. However, despite its widespread use, limitations persist in terms of efficacy and tolerability. The SWOG S0777 trial, a key study evaluating the VRd regimen, reported a median progression-free survival (PFS) of 41 months, with 23% of patients discontinuing treatment due to AEs.^1,2^ Moreover, 33% of patients experienced grade 3 or higher peripheral neuropathy, significantly impacting their quality of life and ability to continue long-term treatment. These challenges highlight the need for optimizing first-line treatment strategies to improve patient outcomes without compromising tolerability.

Peripheral neuropathy, in particular, remains a critical barrier to the continued use of bortezomib in the VRd regimen, often requiring dose reductions or even discontinuation of treatment.^3^ This leads to suboptimal therapeutic outcomes and an unmet need for optimizing first-line treatment strategies to enhance efficacy while reducing toxicity. The development of anti-CD38 monoclonal antibodies, including isatuximab and daratumumab, has offered additional options to address the challenges in the triplet therapy. These anti-CD38 monoclonal antibodies have demonstrated significant efficacies in multiple clinical trials, with manageable AE profiles, making them valuable additions to the VRd backbone. These quadruplet treatment combinations aim to enhance the depth of response, achieve minimal residual disease (MRD) negativity more consistently, and extend PFS and overall survival (OS) beyond what is typically seen with triplet therapies.

Quadruplet regimens have demonstrated their potential to reshape the treatment paradigm for NDMM in both transplantation eligible and ineligible patients. In the IMROZ trial, which enrolled transplant-ineligible patients, isatuximab combined with VRd (Isa-VRd) showed superior efficacy compared to VRd alone, with higher rates of sustained MRD negativity and a substantial reduction in the risk of disease progression or death.^4^ Similarly, the PERSEUS trial, which evaluated the combination of daratumumab with VRd (D-VRd) for both induction therapy before autologous stem cell transplantation (ASCT) and consolidation therapy, further reinforced the value of incorporating monoclonal antibodies into the VRd backbone as first-line treatment, demonstrating a significant improved survival outcomes and MRD negativity rates.^5^ These findings suggest that quadruplet regimens may offer a more effective alternative to triplet therapies which has been demonstrated across all subgroups. The integration of anti-CD38 antibodies into the well-established triplet therapies also aligns with existing clinical workflows and drug availability.

The concept of MRD negativity has gained prominence as a critical endpoint in multiple myeloma treatment, with accumulating evidence suggesting that it is a reliable predictor of long-term outcomes, including PFS and OS.^6,7^ The ability to consistently achieve this outcome has become a central goal of modern myeloma therapy, particularly in the context of optimizing long-term disease management and reducing relapse rates. Given the growing importance of achieving sustained MRD negativity, the addition of monoclonal antibodies in first-line therapy—such as Isa-VRd and D-VRd—has shown significant advantages over the triplet therapies. Furthermore, because of the established safety profiles, the integration of anti-CD38 antibodies into the widely used triplet treatments does not significantly alter the protocol, which lowers the barrier for clinicians to adopt quadruplet therapy in clinical practice.

This consensus paper seeks to explore the evolving role of quadruplet therapies in the management of both transplantation-eligible and transplantation-ineligible NDMM, focusing on their ability to deepen treatment responses, achieve sustained MRD negativity, and improve survival outcomes while managing toxicity. By incorporating evidence from pivotal clinical trials and expert opinions, this consensus aims to provide clear, evidence-based guidance on the integration of quadruplet regimens into standard clinical practice for NDMM. As the treatment landscape continues to evolve, understanding the benefits and challenges of these newer regimens will be key to optimizing patient care and outcomes.

## Materials and Methods

A modified Delphi consensus process was employed to generate statements regarding the use of anti-CD38 monoclonal antibody quadruplet therapy in the treatment of NDMM. The Delphi method is a well-established consensus-based approach for reviewing evidence and synthesizing expert opinion to inform clinical decision-making.^8^ The modified Delphi process in this consensus involved anonymous online voting and in-person meetings by a panel of experts. A 70% agreement threshold was set for consensus generation.^9^ The goal of the panel was to develop consensus recommendations based on recent evidence from pivotal clinical trials and real-world practices, providing clear guidance for optimizing treatment strategies in both transplantation-eligible and transplantation-ineligible patients. Panelists were selected based on their active participation in clinical trials and contributions to the multiple myeloma field, ensuring a diverse range of perspectives. An independent planning committee conducted the selection process, separate from funding bodies, further enhancing the credibility of the panel’s findings. Finally, 16 hematology and oncology experts from the Asia-Pacific region with extensive experience treating NDMM participated in the consensus development. All experts consented to their participation in the development of this consensus statement.

The consensus development process involved a systematic review of the literature. A structured search focusing on peer-reviewed studies was conducted. The Grading of Recommendations Assessment, Development and Evaluation (GRADE) system was used to rate the quality of evidence and the strength of recommendations.^10^ The quality of evidence was rated High (A), Moderate (B), Low, or very low (C) according to the design and limitations of the studies, whereas the strength of recommendations was rated either Strong (1) or Weak (2) based on the benefits and harms of the intervention. Key clinical trials were prioritized due to their significant contributions to understanding the efficacy and safety of quadruplet regimens, particularly those incorporating isatuximab or daratumumab with triplet regimens. In addition to clinical trials, relevant guidelines from authoritative bodies such as the National Comprehensive Cancer Network (NCCN) Guidelines and International Myeloma Working Group (IMWG) were reviewed to ensure that the recommendations were in line with current clinical practice.

A research steering committee jointly reviewed the information and results of the systematic literature review and formulated eight statements for the initial online voting round. These statements encompassed four domains: (i) the benefits and indications for quadruplet therapy in NDMM; (ii) the optimization of quadruplet therapy strategies; (iii) the management and monitoring of potential AEs for quadruplet therapy, and (iv) the impact of quadruplet therapies on tandem stem cell transplantation and maintenance treatment. In October 2024, experts anonymously voted on the statements, expressing their agreement or disagreement and suggesting revisions. An in-person meeting was held in November 2024 to discuss the online voting results and finalize the statements. In December 2024, all participating experts jointly reviewed the consensus document, which underwent multiple rounds of revisions before finalization.

## Results

### Chapter 1: Benefits and Indications of Quadruplet Therapy For NDMM

#### Is anti-CD38-based quadruplet therapy suitable as a first-line treatment for transplantation-ineligible NDMM patients?

##### Statement

Anti-CD38-based quadruplet therapy is suitable for transplantation-ineligible NDMM patients (A1).

##### Discussion

Transplantation-ineligible patients with NDMM often face limited treatment options due to age or comorbidities, making it essential to identify regimens that balance efficacy with tolerability. Traditional triplet therapies have shown significant improvements in survival, but the addition of monoclonal antibodies has the potential to further enhance treatment outcomes.^11–14^

Isa-VRd, a combination of isatuximab, bortezomib, lenalidomide, and dexamethasone, is the only approved quadruplet regimen with a Category 1 recommendation for patients <80 years old who are not frail from the NCCN Guidelines Version 1.2025. The IMROZ study provides robust evidence supporting isatuximab in combination with VRd, abbreviated as Isa-VRd, as a first-line treatment for transplantation-ineligible patients with NDMM.^4^ This multicenter, phase III trial enrolled 446 patients (265 in the Isa-VRd group and 181 in the VRd group), with a median age of 72 years and a median follow-up period of 59.7 months. The study demonstrated a significant 40% reduction in the risk of disease progression or death in the Isa-VRd group compared to VRd alone, and that the estimated PFS at 5 years was significantly longer in the Isa-VRd group compared to the VRd group (63.2% vs. 45.2%, hazard ratio [HR] for disease progression or death: 0.60; 98.5% CI: 0.41–0.88; *P*<0.001), indicating a substantial survival benefit. Furthermore, patients receiving Isa-VRd exhibited a notable improvement in rates of complete response (CR) or better (74.7% vs. 64.1%, *P*=0.01) and higher rates of MRD (55.5% vs. 40.9%, odds ratio [OR] 1.8 [95% CI: 1.23–2.65], *P*=0.003) at a sensitivity threshold of 10^−5^, underscoring the depth of response achieved with this quadruplet regimen.

In addition to efficacy, the safety profile in the IMROZ study was consistent with that in the phase III GMMG-HD7 study of Isa-VRd, in which patients eligible for transplantation were enrolled.^15^ The incidence of serious AEs (SAEs) during treatment and incidence of AEs leading to discontinuation remained comparable in the two groups. This favorable safety profile, combined with the efficacy data, makes Isa-VRd a well-tolerated and effective option as a first-line therapy for transplantation-ineligible patients.

The IMROZ study provides strong evidence for Isa-VRd in transplantation-ineligible patients. Building on these results, the BENEFIT study adds further clarity by comparing Isa-VRd to Isa-Rd in a population aged 65–79 years, with a focus on transplantation-ineligible patients.^16^ This phase III trial enrolled 270 patients, with a median follow-up of 23.5 months. A significant improvement in MRD negativity rates (sensitivity threshold of 10^−5^) was observed in the Isa-VRd group (53%, 95% CI: 44–61) compared to the Isa-Rd group (26%, 95% CI: 19–34) (OR 3.16 [95% CI: 1.89–5.28]; *P*<0.0001), underscoring the advantage of adding bortezomib to Isa-Rd. Additionally, rates of CR or better were substantially higher at 18 months in the Isa-VRd group (58% vs. 33%, OR 2.97 [95% CI: 2–5], *P*<0.0001), as was the MRD negativity rate and CR or better (37% vs. 17%, *P*=0.0003). The result further emphasized the depth of response with the quadruplet regimen. However, with a median follow-up of 23.5 months, PFS and overall survival (OS) data are still immature.

In addition to Isa-VRd, another anti-CD38-based quadruplet therapy, D-VRd, demonstrated superior efficacy compared to VRd in NDMM patients whose transplants were not planned as initial therapy in the phase III CEPHEUS trial.^17^ This latter study enrolled 395 intermediate, fit, transplantation-ineligible, or transplantation-deferred patients with a median age of 70 years. Results showed that the MRD negativity rate (10⁻⁵) was 61% in the D-VRd arm, compared to 39% in the VRd arm, representing a 22% increase in overall MRD negativity with the daratumumab-based quadruplet therapy. Among patients who achieved CR or better, the rate of sustained MRD negativity at 12 months was also significantly higher in the D-VRd arm compared to the VRd arm, at 48.7% and 26.3%, respectively. With a median follow-up of 58.7 months, PFS had not been reached in the D-VRd arm, compared to 52.6 months in the VRd arm. At 54 months, PFS was 68.1% and 49.5% in the D-VRd and VRd arms, respectively, with a statistically significant HR of 0.57.

Patients with high-risk NDMM, particularly those with adverse cytogenetic features such as del17p, t(4;14), t(14;16), and 1q21 amplifications, represent a subset that historically experiences inferior outcomes. The Phase 3 ALCYONE study: Patients were randomized to receive up to nine cycles of either D-VMP or VMP alone. ORR was 90.9% and 73.9%, ≥ CR rate was 46% and 25% in D-VMP group versus VMP, respectively. PFS was 37.3 months and 19.7 months, OS was 82.7 months vs 53.6 months in the two groups, respectively.^18^ Patients with these abnormalities exhibit worse prognoses, making deeper responses and MRD negativity particularly critical in this subset. Addressing the needs of this population requires potent, durable therapies capable of achieving deep remissions.

The GMMG-CONCEPT study, in which 26 transplantation-ineligible, high-risk NDMM patients were enrolled, showed that 54.2% of transplantation-ineligible patients achieved MRD negativity after consolidation therapy with Isa-KRd (KRd: carfilzomib, lenalidomide, and dexamethasone).^19^ Additionally, 46.2% of transplantation-ineligible patients maintained MRD negativity for over a year, indicating the durability of response in high-risk patients. Notably, median PFS had not been reached at a follow-up of 33 months in transplantation-ineligible patients, suggesting that Isa-KRd may offer prolonged disease control in this difficult-to-treat population. The NCCN Guidelines Version 1.2025 have also included the Isa-KRd regimen as a recommended option for use in certain circumstances (Category 2B) for NDMM patients who are ineligible for transplant.

##### Conclusion

The results of IMROZ, BENEFIT, and GMMG-CONCEPT, along with those of the CEPHEUS and ALCYONE trials, highlight the critical role of the quadruplet regimen containing anti-CD38 in optimizing outcomes for NDMM patients who are ineligible for transplant.

##### Level of Consensus

85.7% (12) agree; 14.3% (2) neutral.

Total: 14 voters.

#### Are CD38 monoclonal antibodies plus VRd/KRd suitable as first-line treatments for transplantation-eligible NDMM patients?

##### Statement

CD38 monoclonal antibodies plus VRd/KRd are suitable as a first-line treatment for transplantation-eligible NDMM patients (A1).

##### Discussion

For patients with NDMM who are eligible for transplant, maximizing the depth of response before high-dose chemotherapy and autologous stem cell transplantation (ASCT) is critical for long-term disease control. Quadruplet therapies incorporating monoclonal antibodies offer the possibility of achieving deeper responses compared to traditional triplet regimens.^5,20^ In the NCCN Guidelines Version 1.2025, the D-VRd regimen has been updated from an “other recommended option” to the only preferred regimen for NDMM patients eligible for transplant. Additionally, Isa-VRd has been added as an “other recommended option.”

For transplantation-eligible patients, adding isatuximab to VRd or KRd regimens has demonstrated significant benefits. The GMMG-HD7 study, which included 660 transplantation-eligible patients with a median age of 59 years, investigated the addition of isatuximab to VRd.^15^ Median follow-up periods were 125 days (interquartile range [IQR] 125–131) and 125 days (IQR 125–132) in Isa-VRd and VRd groups, respectively. The study reported a significant increase in MRD negativity rates (at a sensitivity threshold of 10^−5^) following induction therapy, with 50% of patients in the Isa-VRd group achieving MRD negativity compared to 36% in the VRd group (OR 1.82 [95% CI: 1.33–2.48], *P*=0.00017). From a safety perspective, addition of isatuximab to VRd did not lead to new safety signals. In both treatment groups, the rates of discontinuation due to AEs were low. On the other hand, neutropenia was more frequently seen in the Isa-VRd group, which has also been observed in other phase III trials of isatuximab combination therapy in relapsed or refractory multiple myeloma patients.^19,21,22^ These findings suggest that Isa-VRd can induce promising responses before transplant without compromising patient safety, enhancing the efficacy of subsequent treatments such as high-dose melphalan and ASCT.

Another example of quadruplet therapies incorporating isatuximab is the IsKia study, which involved 302 transplantation-eligible NDMM patients (151 patients in both the Isa-KRd and KRd groups), further validated the efficacy of isatuximab.^23^ The addition of isatuximab significantly improved MRD negativity rates at several treatment stages. After consolidation, the MRD negativity rates at a sensitivity threshold of 10^−5^ were 77% in the Isa-KRd group compared to 67% in the KRd group (OR 2.29, *P*=0.049), whereas the MRD negativity rates at a sensitivity threshold of 10⁻^6^ were 67% in the Isa-KRd group compared to 48% in the KRd group (OR 2.29, *P*<0.001). After induction, MRD negativity rates (10⁻^5^) were 45% in the Isa-KRd group compared to 26% in the KRd group (OR 2.34, *P*<0.001), and were 27% versus 14% at a sensitivity threshold of 10⁻^6^ (OR 2.36, *P*=0.004). This benefit was maintained post-transplant, where 64% of Isa-KRd patients achieved MRD negativity (10⁻^5^) compared to 49% in the KRd group (OR 1.93, *P*=0.006), and were 52% versus 27% at a sensitivity threshold of 10⁻^6^ (OR 3.01, *P*<0.001). These profound MRD responses underscore the ability of Isa-KRd to provide durable disease control.

Moreover, Isa-KRd has shown promise in overcoming the challenges associated with high-risk disease.^19^ The IsKia study addressed the efficacy of Isa-KRd in high-risk NDMM, revealing that MRD negativity rates were comparable across high-risk and standard-risk patients.^23^ Specifically, MRD negativity rates of the Isa-KRd group at a sensitivity threshold of 10^-5^ were 76% in high-risk patients and 77% in double-hit patients, closely mirroring the rates seen in standard-risk patients (79%). In comparison, the KRd group demonstrated a notable decline in MRD negativity in high-risk (58%) and double-hit (53%) patients, which were inferior to the one in standard-risk patients (70%), highlighting Isa-KRd’s ability to achieve deeper responses in those with aggressive disease. Similarly, the GMMG-CONCEPT study which included 99 transplantation-eligible, high-risk NDMM patients, further reinforces Isa-KRd’s role in this population.^19^ The interim analysis showed that 67.7% of transplantation-eligible patients achieved MRD negativity after consolidation therapy. Moreover, 62.6% of transplantation-eligible patients maintained MRD negativity for over a year, and that the median PFS had not been reached at a follow-up of 44 months in these patients, suggesting that Isa-KRd may offer prolonged disease control in this difficult-to-treat population.

These findings underscore the efficacy of Isa-KRd in achieving sustained responses across various high-risk subgroups. Its ability to deliver high MRD negativity rates, regardless of transplant eligibility, highlights its potency as a first-line treatment option for high-risk NDMM. Additionally, the safety profile of Isa-KRd remained favorable, with a toxicity profile similar to that in previous reports.^19,23^ Common AEs such as hematologic toxicities (e.g., neutropenia and thrombocytopenia) were manageable and the safety profile was similar to that seen in the KRd group. Given the high MRD negativity rates across various subgroups and its efficacy in maintaining these responses, Isa-KRd is emerging as a highly effective regimen for high-risk NDMM, regardless of transplant eligibility. Its tolerability and safety profile further support its use in a broad patient population, providing a potent option for achieving long-term disease control in high-risk individuals.

Compared to isatuximab-based quadruplet therapies, daratumumab-based quadruplet regimens had demonstrated significant efficacy in transplantation-eligible NDMM patients through multiple controlled clinical studies. The phase III PERSEUS trial demonstrated that the combination of daratumumab with VRd (D-VRd), improves PFS, and rate of CR or better, as well as MRD negativity compared to the VRd group.^24^ The D-VRd group demonstrated significantly higher rates of CR or better (87.9% vs. 70.1%, *P* < 0.001) and MRD negativity (75.2% vs. 47.5%, *P* < 0.001) compared to the VRd group. At a median follow-up of 47.5 months, the estimated 48-month PFS was 84.3% in the D-VRd group and 67.7% in the VRd group, with a statistically significant HR of 0.42. Moreover, the benefits of daratumumab were consistent across various patient subgroups, including those with International Staging System stage III disease or high cytogenetic risk. Regarding safety, the proportion of patients experiencing grade 3-4 AEs was 91.5% in the D-VRd group, higher than the 85.6% observed in the VRd group. The most common grade 3 or higher AEs were hematologic abnormalities, including neutropenia (62.1% in the D-VRd group vs. 51.0% in the VRd group) and thrombocytopenia (29.1% vs. 17.3%, respectively). The incidence of SAEs was also higher in the D-VRd group (57.0%) compared to the VRd group (49.3%). However, the treatment discontinuation rate due to AEs was lower in the D-VRd group (8.8%) than in the VRd group (21.3%). Overall, the safety profile was consistent with the known safety profiles of daratumumab and of VRd in this patient population. Besides the PERSEUS study, the phase III CASSIOPEIA trial showed that adding daratumumab to bortezomib, thalidomide, and dexamethasone (D-VTd) improved MRD negativity rates (64% vs. 44%, *P*<0.0001).^20^ The GRIFFIN study also demonstrated enhanced MRD negativity (64% vs. 30%) with D-VRd in transplantation-eligible patients.^5^ In the final analysis of the GRIFFIN study, at a median follow-up of 49.6 months (IQR 47.4–52.1), D-VRd demonstrated superior efficacy, significantly improving the rate of stringent complete response (67% (67 of 100) vs. 48% (47 of 98); odds ratio 2.18 (95% CI: 1.22–3.89), p = 0.0079). The 4-year PFS was 87.2% (95% CI: 77.9–92.8) with D-VRd, compared to 70.0% (95% CI: 55.9–80.3) with VRd. The risk of disease progression or death was significantly reduced with D-RVd, with a hazard ratio (HR) of 0.45 (95% CI: 0.21–0.95, p = 0.032).^25^ Based on the impressive benefits, with manageable increased toxicities, the Dara-VRd regimen has been included in the NCCN Guidelines Version 1.2025 for primary treatment of transplant-eligible NDMM.

On the other hand, results from the phase II MASTER trial, which evaluated D-KRd in patients with NDMM with enrichment for HRCAs.^26^ MRD negativity (10–5) rates were comparable across different risk group: 78% in patients with no HRCAs, 86% in patients with one HRCA and 79% in patients with two or more HRCAs. The 3-year PFS among patients with no, one, or two or more chromosome abnormalities were 88% (95% CI: 78–95), 79% (95% CI: 67–88), and 50% (95% CI: 30–70), respectively. A post hoc analysis of patients with high-risk cytogenetic abnormalities in the MASTERS and GRIFFIN studies were performed.^27^ At the median follow-up (MASTER: 31.1 months; GRIFFIN: 49.6 months for randomized patients), minimal residual disease (MRD) negativity rates, as assessed by next-generation sequencing (10⁻⁵), were 80.0%, 86.4%, and 83.3% for patients with 0, 1, or ≥2 high-risk cytogenetic abnormalities (HRCAs) treated with D-KRd, and 76.1%, 55.9%, and 61.5% for those treated with D-RVd. Progression-free survival (PFS) was comparable between studies and was superior in patients with 0 or 1 HRCA compared to those with ≥2 HRCAs. The 36-month PFS rates were 89.9%, 86.2%, and 52.4% for D-KRd, and 96.7%, 90.5%, and 53.5% for D-RVd. Similarly, findings from the GMMG-CONCEPT and IsKia trials suggest that isatuximab may also offer robust disease control in high-risk NDMM patients, as evidenced by Isa-KRd demonstrating sustained MRD negativity (10⁻⁵ and 10⁻⁶) in high-risk subgroups.^19,23^

##### Conclusion

The findings in these pivotal trials emphasize that anti-CD38 monoclonal antibody-based quadruplet regimens significantly improve outcomes across transplantation-eligible and -ineligible populations. In addition, anti-CD38 monoclonal antibodies may offer superior efficacy in high-risk NDMM, further supporting the growing role of quadruplet therapies in NDMM management.

##### Level of Consensus

92.9% (13) agree; 7.1% (1) neutral.

Total: 14 voters.

### Chapter 2: Optimization of Treatment Strategy

#### How can treatment strategies be optimized for NDMM to maximize outcomes?

##### Statement

For NDMM patients less than 80 years old who are not frail and those with mild renal impairment (e.g., estimated glomerular filtration rate [eGFR] between 60 and 89 mL/min/1.73 m²), quadruplet therapy is the preferred regimen (A1).

##### Discussion

In the phase III MAIA study, the combination of daratumumab, lenalidomide and dexamethasone (Dara-Rd) has demonstrated improved median PFS comparing to lenalidomide and dexamethasone (Rd) group (median, 61.9 vs 34.4 months; HR, 0.55; 95% CI, 0.45-0.67; P<0.0001), with comparable safety profiles. Besides, the median overall survival was not reached in Dara-Rd group, while 65.5 months in patients in the Rd group (HR, 0.66; 95% CI, 0.53-0.83; P=0.0003). Besides, the overall response rate (ORR) was significantly higher for Dara-Rd compared to Rd (92.9% vs. 81.6%; P < 0.0001), along with higher rates of MRD negativity (32.1% vs. 11.1%; P < 0.0001) and sustained MRD negativity for ≥12 months (18.8% vs. 4.1%; P < 0.0001).^28,29^ A subgroup analysis of the MAIA study by frailty status categorized based on age, Charlson Comorbidity Index (CCI), and Eastern Cooperative Oncology Group (ECOG) performance status was performed. After a 36.4-month median follow-up, the triplet therapy Dara-Rd demonstrated not only a significant PFS benefit over Rd in transplantation-ineligible NDMM patients, but also including those classified as frail (NR vs 30.4 months; HR, 0.62; 95% CI, 0.45–0.85; P =0.003). Dara-Rd achieved higher overall response rates (ORR) (87.2% vs. 78.1%, P = 0.0265) as well as higher ≥CR and MRD negativity rates (23.8% vs. 10.1%) compared to Rd in frail patients. Median time to ≥CR was also shorter with D-Rd versus Rd (10.6 vs 11.5 months). While grade 3/4 adverse events were more frequent in the frail subgroup (94.6% with D-Rd vs. 89.2% with Rd), with neutropenia being the most common (57.7% vs. 33.1%), the rate of treatment discontinuations due to AEs was lower with D-Rd compared to Rd (10.1% vs. 19.3%). Despite the higher incidence of adverse events, the overall safety profile of D-Rd remained manageable.^30^ Based on the MAIA results, Dara-Rd has been listed as one of the preferred initial treatment regimens for transplantation-ineligible NDMM in the NCCN Guidelines Version 1.2025.

The rapid development of quadruplet regimens has significantly broadened the treatment landscape for NDMM. Traditional triplet therapies have shown significant improvements in survival; however, these advancements offer clinicians greater flexibility in tailoring treatment strategies to patient-specific characteristics, thereby optimizing therapeutic outcomes. Real-world evidence indicates that quadruplet regimens, especially those incorporating monoclonal antibodies, have become increasingly standard in clinical practice due to their ability to achieve deeper responses and higher rates of MRD negativity.^31^ While traditional doublet and triplet therapies remain effective, quadruplet regimens incorporating monoclonal antibodies like isatuximab or daratumumab have shown enhanced efficacy, offering deeper responses and higher MRD negativity rates.^15,19,23,32^ Importantly, this evolution in treatment allows even patients who were previously considered suitable only for doublet or triplet therapy—such as older patients or those with multiple comorbidities—to achieve better outcomes, as the tolerability of quadruplet regimens has improved. Here, we outline an optimization strategy, including the selection criteria for doublet, triplet, and quadruplet regimens, to guide clinical decision-making.

Given the variability in patients’ health status, age, comorbidities, and disease characteristics, choosing an appropriate regimen is crucial. Real-world data support the necessity of individualizing treatment plans and making dose adjustments in quadruplet regimens, particularly for older or frail patients.^33^ Importantly, as mentioned in the most updated NCCN guidelines: Multiple Myeloma (version 1.2025), Isa-VRd is the preferred regimen for transplantation-ineligible patients less than 80 years old who are not frail. This adaptability allows a broader range of patients to benefit from the enhanced efficacy of quadruplet therapy while minimizing toxicity. A comprehensive assessment of each patient’s fitness, cytogenetic risk, and treatment goals is necessary for optimizing therapy selection. The key patient characteristics suitable for each regimen are summarized in **Table 1**.

**Table 1.** Relation of patient characteristics for treatment regimens

*Regimen*	*Patient Characteristics*
Doublet Therapy	• Elderly patients (age ≥80) • Significant comorbidities or frailty • Limited tolerance to intensive therapy due to poor performance status • Severe renal impairment (e.g., severe reduction in eGFR)
Triplet Therapy	• Patients aged ≥80 with moderate fitness • Some comorbidities but manageable • High-risk cytogenetic abnormalities but unfit for quadruplet regimens • Moderate renal impairment (e.g., moderate reduction in eGFR)
Quadruplet Therapy	• Not frail patients (age < 80) • Transplantation-eligible or -ineligible with good performance status • High-risk cytogenetic abnormalities • Older patients with adequate fitness and manageable comorbidities • Mild renal impairment (e.g., mild reduction in eGFR)

Assessing patient fitness using validated tools, such as the IMWG frailty score, is recommended to identify those who may benefit most from intensive quadruplet regimens.^34^ The IMWG frailty score is considered as the gold standard and classifies patients into three categories: fit, intermediate fit, and frail, which helps predict mortality and the risk of toxicity in first-line treatment.^35^ In addition, renal impairment is a common issue in multiple myeloma. The IMWG has provided updated guidelines for the management of multiple myeloma-related renal impairment.^36^ Serum creatinine, eGFR, and free light chains are key measurements used to assess renal status. The eGFR is an indicator for kidney disease monitoring and management.^37^ Incorporating these objective assessments allows clinicians to balance treatment efficacy with potential toxicity, particularly in patients who were historically limited to less intensive regimens. In addition, for patients with impaired renal function (e.g., patients with eGFR < 30 mL/min/1.73 m^2^, or patients under dialysis), the immunomodulatory medication lenalidomide may be substituted with thalidomide, pomalidomide, or cyclophosphamide. For patients with eGFR < 30 mL/min/1.73 m^2^, the daily use of lenalidomide should be further adjusted, as recommended by the NCCN guidelines: Multiple Myeloma (version 1.2025).

Quadruplet regimens, such as Isa-VRd, Isa-KRd, and D-VRD, have demonstrated higher MRD negativity rates, improved PFS, and OS in both transplantation-eligible and transplantation-ineligible patients. For instance, the IMROZ trial reported a substantial improvement in the depth of response in patients treated with Isa-VRd.^4^ It is noteworthy that patients up to 80 years of age with a median age of 72 years, were included in IMROZ, and that 26.0% (69/265) of the Isa-VRd group were aged 75–80 years, suggesting that Isa-based quadruplet therapy can be beneficial even for very elderly (≥75 years) NDMM patients. Furthermore, in the IMROZ trial, 29% of patients were classified as frail based on age, comorbidities, and Eastern Cooperative Oncology Group performance status, with 28% and 32% in the Isa-VRd and VRd arms, respectively. Frail patients treated with Isa-VRd had a median treatment duration of 31.5 months compared to 23.7 months for those on VRd. Isa-VRd significantly improved progression-free survival (PFS) in frail patients (HR=0.584; 95% CI: 0.340–1.004; p=0.0516) and showed higher rates of complete response or better (61.6% vs 50.9%) and minimal residual disease negativity (50.7% vs 22.8%) compared to VRd. Safety outcomes were favorable, with fewer frail patients discontinuing treatment in the Isa-VRd arm (29.2% vs 35.1%) and no increase in grade ≥3 infections. Similarly, the BENEFIT study which included transplantation-ineligible patients aged 71-76 years, observed a significant improvement in MRD negativity rates in the Isa-VRd group, further suggesting that novel quadruplet-based strategies may benefit the elderly population.^16^ Furthermore, the GMMG-CONCEPT study enrolled 26 transplantation-ineligible, high-risk NDMM patients with a median age of 74 years (64–87) and demonstrated sustained MRD negativity after consolidation therapy and maintenance with Isa-KRd.^19^ In CEPHEUS trial, 55.3% patients in D-VRd group are ≥70 years, elderly patients treated with D-VRd are more likely to achieved MRD negativity compared with VRd group (ORR, 2.06, 95% CI, 1.2-3.53). 64% patients in D-VRd group are ECOG ≥1, patients treated with D-VRd are more likely to achieved MRD negativity compared with VRd (ORR, 2.88, 95% CI, 1.71-4.87). However, in the CEPHEUS trial, the frail patients were excluded during enrollment.^38^ Therefore, the safety and efficacy of daratumumab in quadruplet regimens for frail patients still require further investigation. Collectively, these studies support the application of quadruplet regimens with anti-CD38 monoclonal antibodies in the elderly NDMM population; whereas in frail patients, the tolerability and efficacy of the isatuximab-based quadruplet regimen have been demonstrated in clinical trials.

Apart from patients’ health status and age, comorbidities and disease characteristics are also crucial in choosing an appropriate regimen. The improved tolerability of quadruplet regimens has broadened their applicability to patients who were once deemed unsuitable for intensive therapy. This includes not only older patients but also those with comorbidities who were historically managed with doublet or triplet therapies due to concerns about toxicity. Recent clinical data support the use of quadruplet regimens in these populations, offering them the potential for deeper remissions and prolonged disease control. For example, findings from the GMMG-CONCEPT, IsKia, MASTER, and GRIFFIN trials showed that anti-CD38 monoclonal antibodies may offer potential disease control in high-risk NDMM patients.^19,23,27^

The optimal strategy involves a careful evaluation of patient characteristics, including age, fitness, cytogenetic risk factors, and individual treatment goals. Recent clinical data and real-world studies suggest a gradual shift towards using quadruplet therapies in NDMM patients who were previously considered unsuitable for intensive treatment, owing to improved tolerability and management of AEs.^39^ Clinicians should aim to maximize efficacy while minimizing toxicity, choosing quadruplet regimens for fit patients and those who can now tolerate these intensive therapies due to improved safety profiles. For older patients or those with high-risk cytogenetic abnormalities, dose modifications and proactive management of AEs allow for a safer application of these advanced therapeutic regimens. In contrast, for patients with significant frailty or multiple comorbidities who still might not tolerate intensive treatment, a doublet or triplet approach remains a viable option, with potential dose modifications to enhance tolerability.

**Figure 1.**
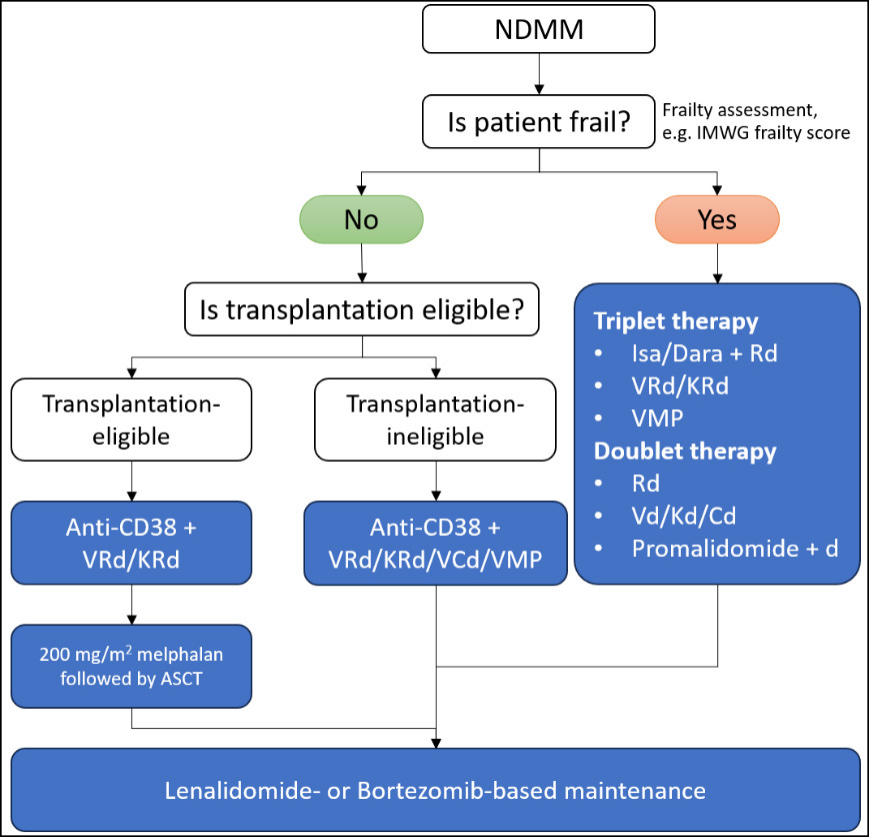
The proposed algorithms for newly diagnosed multiple myeloma. Anti-CD38, anti CD38 monoclonal antibodies; ASCT, autologous stem cell transplantation; Cd, cyclophosphamide + dexamethasone; d, dexamethasone; Dara, daratumumab; IMWG, International Myeloma Working Group; Isa, isatuximab; Kd, carfilzomib + dexamethasone; KRd, carfilzomib + lenalidomide + dexamethasone; Vd, bortezomib + dexamethasone, VRd, bortezomib + lenalidomide + dexamethasone.

While quadruplet regimens offer deeper responses, they also come with potential limitations, including increased toxicity and cost. However, the recent improvements in the tolerability profile of quadruplet therapies have made them a viable option for a broader range of patients than previously considered. In summary, quadruplet regimens represent a transformative step forward in NDMM treatment, allowing for more tailored and effective therapeutic strategies. These regimens have not only shifted the treatment goal towards achieving MRD negativity but have also made it possible for older and frailer patients to benefit from deeper responses previously unattainable with doublet or triplet therapies. Ongoing monitoring and individualizing treatment plans, particularly in the context of real-world patient diversity, are essential to ensure that patients achieve optimal outcomes while managing the potential risks associated with more intensive quadruplet regimens.^33^ The proposed algorithms for NDMM are shown in **Figure 1**.

##### Conclusion

Patients under 80 years old with adequate health condition and manageable comorbidities are ideal candidates for the quadruplet regimens. For frail or comorbid patients, triplet or doublet therapies remain suitable alternatives. Personalized treatment strategies, guided by frailty and renal assessments, are essential to maximize outcomes while minimizing toxicity. These advancements mark a shift toward deeper, more durable responses and broader accessibility to intensive therapies in NDMM management.

##### Level of Consensus on that quadruplet therapy is the preferred regimen for NDMM patients less than 80 years old who are not frail

78.6% (11) agree; 21.4% (3) neutral.

Total: 14 voters.

##### Level of Consensus on that quadruplet therapy is preferred for NDMM patients with mild renal impairment (e.g., eGFR between 60 and 89 mL/min/1.73 m²)

92.9% (13) agree; 7.1% (1) neutral.

Total: 14 voters.

### Chapter 3: Adverse Event Monitoring and Management

#### What are the common AEs of quadruplet regimens and how should we manage them?

##### Statement

Hematologic toxicities are among the most common AEs in patients undergoing quadruplet therapy. Management involving routine complete blood count (CBC) monitoring, dose adjustments, or granulocyte colony-stimulating factor (G-CSF) support are recommended (A1).

##### Discussion

From a safety perspective, addition of isatuximab to VRd did not lead to new safety signals. Neutropenia was more frequently seen in the Isa-VRd group, which has also been observed in other phase III trials of isatuximab combination therapy in relapsed or refractory multiple myeloma patients.^19,21,22^ Additionally, the safety profile of Isa-KRd remained favorable, with a toxicity profile similar to that in previous reports.^19,23^ Common AEs such as hematologic toxicities (e.g., neutropenia and thrombocytopenia) were manageable and the safety profile was similar to that seen in the KRd group. In parallel, daratumumab also demonstrated a similarly manageable safety profile in quadruplet regiments. Clinical data from the GRIFFIN and ALCYONE trials revealed a safety profile characterized by manageable AEs, with a profile of most frequently observed AEs, similar to isatuximab-based quadruplet therapy.^5^

Building on these findings, the introduction of quadruplet regimens like Isa-VRd, Isa-KRd, Dara-VRd and Dara-KRd has redefined the management of NDMM. However, the use of these regimens is associated with a range of AEs that vary based on patient eligibility for transplant. Clinical trial data from the IMROZ, GMMG-HD7, GMMG-CONCEPT, BENEFIT, GRIFFIN and ALCYONE studies offer valuable insights into the incidence and management of these AEs across different patient populations. A recent meta-analysis of phase II and phase III clinical trials comparing the risk of AEs between quadruplet and triplet regimens revealed that AEs were more frequent with the quadruplet regimens but were predominately mild.^40^ Grade ≥3 AEs that were more common with quadruplet regimens were infections (relative risk [RR] 1.34; 95% CI: 1.17– 1.54) and thrombocytopenia (RR 1.39; 95% CI: 1.12–1.74).^40^ Notably, the analysis did not show an increased risk for anemia, neuropathy, or pneumonia at any grade. The approaches to managing the common AEs and the incidence observed in patients treated with quadruplet therapy in clinical trials are summarized in **Table 2**.

**Table 2.** Common AEs and managements

*Adverse events*	*Incidence*	*Approach*
Neutropenia	≥ Grade 3 in transplantation-ineligible: 28%-54.4%; ≥ Grade 3 in transplantation-eligible: 23.3%-62.1%	Regular complete blood count (CBC) monitoring is essential. In cases of grade 3 or 4 neutropenia, dose modification or the administration of granulocyte colony-stimulating factor (G-CSF) is recommended to preserve sufficient neutrophil levels and mitigate the risk of infection.
Infections	≥ Grade 3 in transplantation-ineligible: 22%-44.9%; ≥ Grade 3 in transplantation-eligible: 12%-35.3%	The prophylactic administration of antibiotics, commencing at the induction phase, has been shown to decrease the occurrence of grade 3 or higher infections.
Cardiovascular	≥ Grade 3 in transplantation-ineligible: 8%-20%; ≥ Grade 3 in transplantation-eligible: 2.1%-9%	• Before initiating treatment, a baseline cardiac assessment should be performed, including echocardiography and cardiac biomarkers such as troponin and NT-proBNP. • During treatment, patients should be monitored for blood pressure, symptoms of dyspnea, chest pain, edema, or fatigue, to detect potential cardiac dysfunction. If cardiac dysfunction is suspected, further echocardiography should be performed. If the left ventricular ejection fraction (LVEF) decreases by more than 10% and falls below the normal range, treatment with angiotensin- converting enzyme (ACE) inhibitors or beta-blockers may be considered.
Peripheral sensory neuropathy	≥ Grade 3 in transplantation-ineligible: 1.4%-27%; ≥ Grade 3 in transplantation-eligible: 2.1-7%	Regularly assess patients for symptoms of neuropathy and adjust the bortezomib dosing regimen as needed. Administering normal saline infusion and other supportive measures (such as the use of emollients) during bortezomib treatment may help prevent or alleviate neuropathy. For patients experiencing Grade 3 neuropathy, consider pausing bortezomib therapy until symptoms improve.

##### Hematologic Abnormalities

Hematologic toxicities, including neutropenia, lymphopenia, anemia, and thrombocytopenia, are among the most common AEs in patients undergoing quadruplet and triplet therapy. Data from the IMROZ trial, which focused on transplantation-ineligible patients, indicated that grade 3 or 4 neutropenia occurred in 54.4% of patients treated with Isa-VRd.^4^ Similarly, the BENEFIT study, targeting older patients aged 65–79 years, reported a high incidence of hematologic AEs, with grade 3 or 4 neutropenia affecting 40% of patients.^32^ Thrombocytopenia was also found with a higher incidence in the Isa-VRd group compared with the Isa-Rd group. In contrast, in the GMMG-HD7 trial, which included transplantation-eligible patients, the occurrence of hematologic abnormalities was somewhat lower, with grade 3 or 4 neutropenia reported in 23% of patients receiving Isa-VRd.^15^ This difference may be attributed to the younger (median age 59, range 54–64 years), generally healthier transplantation-eligible population, which often exhibits better tolerance to intensive therapy. Similarly, in daratumumab-based therapies, the ALCYONE trial targeting older patients aged over 65 years revealed that neutropenia was the most common grade 3 or 4 AE during treatment cycles (40% in the daratumumab plus bortezomib, melphalan, and prednisone (D-VMP) group and 39% in the VMP group).^5^ In the GRIFFIN study, grade 3 or 4 neutropenia was observed in the D-RVd arm with an occurrence of 41.1% among the grade 3 or 4 AEs.^5^ These findings demonstrated that hematological abnormalities are among the most common AEs in CD38-based quadruplet therapies.

Management of hematologic toxicities involves routine CBC monitoring, particularly during the induction phase. For patients with grade 3 or 4 neutropenia, dose adjustments or G-CSF support is recommended to maintain adequate neutrophil counts and reduce the risk of infections.^41^ While G-CSF prophylaxis is not routinely used in multiple myeloma, it is advised for patients undergoing regimens with a high (≥ 20%) risk of febrile neutropenia, those with intermediate risk (10–20%) who have additional factors (e.g., age > 65 years, frailty), or those on treatments with a neutropenia rate over 50% (e.g., lenalidomide-based combinations).^42^ G-CSF may also be considered for patients with aggressive disease to prevent treatment delays or reactively for those who develop neutropenia during therapy.

##### Infections

Increased infection rates are another concern in quadruplet regimens. In the ALCYONE trial, patients treated with D-VMP experienced a higher rate (22%) of grade 3 or higher infections such as pneumonia, as compared to the VMP group (15%). A higher rate of infection was also reported in the D-RVd group compared to the RVd group in the GRIFFIN trial. Similarly, in the IMROZ trial, the incidence of infections of grade 3 or higher was 44.9% in the Isa-VRd group compared to the VRd group (38.1%). It is shown that antibiotic prophylaxis starting at induction reduces the incidence of infection of grade 3 or higher.^4^ Furthermore, infections were observed more frequently in the Isa-VRd regimen compared to the Isa-Rd (47% vs. 39%) in the BENEFIT trial.^32^ In the GMMG-HD7 trial, however, in which younger transplantation-eligible patients were included, the incidence of grade 3 or 4 infections was 12%.^15^ The data suggest that the addition of isatuximab to quadruplet regimens, such as Isa-VRd, is associated with an increased incidence of infections, particularly of grade 3 or higher, as observed in both the BENEFIT and IMROZ trials. However, the reduced rate of severe infections in the GMMG-HD7 trial, which included a younger, transplantation-eligible population, indicates that patient age and fitness may play a significant role in infection risk. These findings underscore the importance of patient selection and prophylactic interventions, such as antibiotic prophylaxis starting at induction, to mitigate infection-related complications during treatment.

The management of viral (e.g., herpes) and bacterial (e.g., *Staphylococcus, Escherichia coli*) infections are important for multiple myeloma patients on quadruplet regimens.^43^ To reduce infection risks, the IMWG recommends antibacterial, antifungal, and antiviral prophylaxis.^44^ Polyclonal immunoglobulin (Ig) replacement may be considered for patients with IgG less than 400 mg/dL or specific antibody failure.^45^ Antifungal prophylaxis should be used carefully because of potential drug interactions, particularly with lenalidomide, whose exposure increases when combined with itraconazole, heightening toxicity risk.^46^ Monitoring lenalidomide levels and creatinine clearance is recommended. Although pomalidomide has fewer interaction concerns with inhibitors like ketoconazole, its use still requires oversight. Additionally, vaccination is advised as per guidelines from the European Myeloma Network (EMN), Centers for Disease Control, Infectious Diseases Society of America, and the NCCN to prevent infections.^45,47^ These tailored prophylactic strategies are key to managing infection risks in multiple myeloma patients undergoing quadruplet therapy.

##### Cardiovascular AEs

When using Isa-KRd, particularly in the context of the GMMG-CONCEPT trial, cardiovascular toxicities were observed more frequently. In this trial, transplantation-eligible patients experienced grade 3 or higher cardiovascular AEs at a rate of 2.1%, whereas 20% of transplantation-ineligible patients experienced cardiovascular AEs of grade 3 or higher,^19^ emphasizing the need for vigilant monitoring. The relatively high incidence of cardiovascular events in the transplantation-eligible cohort could be related to the use of carfilzomib, which has known associations with cardiovascular risk. Conversely, the BENEFIT study, which utilized bortezomib instead of carfilzomib, reported a lower incidence (8%) of cardiovascular AEs in the transplantation-ineligible population.^16^

Management strategies for cardiovascular AEs include pre-treatment cardiac evaluation with echocardiography and cardiac biomarkers (e.g., troponin, NT-proBNP) to establish a baseline. During treatment, patients should be monitored for symptoms like dyspnea, chest pain, edema, or fatigue to detect potential cardiac dysfunction. Blood pressure should be assessed regularly, especially for patients on Isa-KRd. If cardiac dysfunction is suspected, further echocardiography is recommended. A decline in left ventricular ejection fraction (LVEF >10% below normal) may require angiotensin-converting enzyme (ACE) inhibitors or beta-blockers.^48^ For carfilzomib-related grade 3 or higher cardiac AEs, the EMN advises suspending the drug, administering fluids, and considering a lower dose if therapy is restarted, along with regular cardiac monitoring. Preventive measures include cardioprotective drugs such as ACE inhibitors (e.g., enalapril), angiotensin receptor blockers (e.g., candesartan), and specific beta-blockers (e.g., carvedilol). Transplantation-ineligible patients require a conservative approach with frequent monitoring due to compounded cardiovascular risks.^49–51^

##### Other AEs

Hypogammaglobulinemia is another significant AE associated with both daratumumab and isatuximab. In patients receiving daratumumab, hypogammaglobulinemia was linked to an increased risk of infections, necessitating close monitoring of IgG levels. Intravenous Ig (IVIG) replacement therapy may be considered for patients with severe IgG depletion or recurrent infections.^45^ CD38-based therapies have also been reported to be associated with infusion-related reactions (IRRs). These reactions are typically mild and occur during the first infusion, presenting as symptoms such as fever, chills, and respiratory distress and are generally manageable with pre-infusion medications. For example, in the GRIFFIN trial, IRRs occurred in 72.4% patients and were generally mild, with most patients experiencing them during their first infusion. Peripheral neuropathy is another concern, particularly with the use of bortezomib in regimens like Isa-VRd. In the context of peripheral neuropathy concerns that may lead to treatment discontinuation, adjusting the bortezomib dose from twice-weekly to once-weekly, with regular assessment for neuropathy can still make quadruplet therapy with Isa-VRd a viable option. The BENEFIT study supports this approach, with subgroup analysis showing that Isa-VRd with a weekly bortezomib schedule possessed consistent MRD improvement versus Isa-Rd across clinically relevant subgroups, including patients with International Staging System (ISS) stage III disease, with comorbidities, or high-risk cytogenetics.^16^ This flexibility is especially relevant for patients at risk of adverse effects like peripheral neuropathy, as it helps mitigate those risks without compromising treatment outcomes. Gastrointestinal symptoms, including nausea and diarrhea, should be managed using supportive care measures like antiemetics and adequate hydration. For regimens involving monoclonal antibodies such as isatuximab or daratumumab, infusion-related reactions are common, particularly during the initial administration. Close monitoring during infusions is also essential to ensure early detection and management of any IRRs.

##### Conclusion

Hematologic toxicities, particularly neutropenia and thrombocytopenia, are among the most frequent AEs in patients undergoing CD38-based quadruplet therapy for NDMM. Clinical data from trials highlight the prevalence of these AEs, with grade ≥3 neutropenia affecting up to 62% of patients. These events are manageable with proactive strategies, including routine CBC monitoring, dose adjustments, and the use of G-CSF to maintain adequate neutrophil counts and reduce infection risks. While infections and thrombocytopenia also pose significant concerns, the careful application of preventive measures, such as antibiotic prophylaxis and supportive care, ensures patient safety.

##### Level of Consensus

100.0% (14) agree.

Total: 14 voters.

#### How do quadruplet regimens impact infection susceptibility and what preventive measures can we take?

##### Statement

Quadruplet regimens may increase the infection susceptibility. Therefore, prophylactic antiviral (e.g., acyclovir or valacyclovir) and antibacterial agents (e.g., trimethoprim-sulfamethoxazole), and vaccination against influenza and pneumococcal infections are advised prior to initiating quadruplet therapy (A1).

##### Discussion

Quadruplet therapy regimens, while offering enhanced efficacy in treating NDMM, can significantly increase patients’ susceptibility to infections due to their immunosuppressive effects. The addition of monoclonal antibodies like isatuximab or daratumumab further compounds this risk, as they target CD38 on plasma cells, leading to a reduction in normal B-cell and plasma cell function. This immunomodulatory action can result in lymphopenia and hypogammaglobulinemia, which diminish the body’s ability to fend off viral, bacterial, and fungal pathogens.

In the previous isatuximab- and daratumumab-based quadruplet trials,^4,11–15^ infections were one of the most frequently reported AEs. Patients undergoing quadruplet therapy particularly exhibited a higher incidence of viral reactivation as well as bacterial infections. Transplantation-ineligible patients, often older and with more comorbidities, were at an even greater risk, emphasizing the need for vigilant preventive measures and management strategies in these individuals. It is worth noting that patients receiving anti-CD38 monoclonal antibody therapy are at heightened risk of infection with varicella-zoster virus (VZV).^52^ As mentioned in the NCCN guidelines: Prevention and Treatment of Cancer-Related Infections, Other infection concerns include *Listeria*, hepatitis B virus (HBV), cytomegalovirus (CMV), *Pneumocystis jirovecii* pneumonia (PJP) and *Cryptococcus*. The NCCN panel recommends antiviral prophylaxis, PJP prophylaxis, and monitoring for drug-induced neutropenia. The European Society of Clinical Microbiology and Infectious Diseases (ESCMID) study group also provides guidance on infectious risks in patients receiving CD38-directed therapies.^52^

##### Preventive Measures

Prophylactic antiviral (e.g., acyclovir or valacyclovir) and antibacterial agents (e.g., trimethoprim sulfamethoxazole) should be considered.^53^ Additionally, vaccination against influenza and pneumococcal infections is advised prior to initiating therapy. The ESCMID Study Group indicated that when feasible, clinicians should encourage seasonal influenza vaccination in patients receiving quadruplet therapies.^52^ In addition to vaccinating the patients themselves, community immunization is strongly advocated, where close contacts, including family members living with the patient, are also vaccinated.^54^ The “cocoon vaccination strategy” reduces the overall risk of viral transmission to patients undergoing therapies that suppress immune function. The multifaceted approach to mitigate the risk of infections is summarized in **Table 3**.

**Table 3.** Approaches for prophylaxis of infections

*Preventive Measures*	*Approach*
Antiviral Prophylaxis	• Prophylactic antiviral agents, such as **acyclovir** or **valacyclovir**, should be administered to prevent **herpes zoster reactivation**, which has been observed at higher rates in patients receiving isatuximab or daratumumab-based quadruplet therapies. • Prophylaxis should be continued throughout the course of therapy and for several months after its completion to reduce the risk of late-onset viral infections. • For patients with suspected CMV-related disease (eg, colitis, pneumonitis, hepatitis) or otherwise unexplained fever and/or cytopenias, the viral load of CMV should be monitored by PCR-based methods.
Antibacterial Prophylaxis	• For patients at high risk of ***Pneumocystis jirovecii* pneumonia (PJP)**, such as those with significant neutropenia or lymphopenia, **trimethoprim-sulfamethoxazole** is recommended as a prophylactic measure. • In cases where patients cannot tolerate this regimen, alternatives like **atovaquone** or **dapsone** may be considered.
Vaccinations	• Vaccination is a critical component of infection prevention. Patients at higher risk of infection are advised to receive vaccines against **influenza** and **pneumococcal infections** prior to the initiation of quadruplet therapy. • Live vaccines should be avoided during treatment due to the compromised immune status of patients. In some cases, **booster doses** of inactivated vaccines may be warranted to enhance protective immunity.
Immunoglobulin levels monitoring	• Regular assessment of immunoglobulin (Ig) G level is essential, as hypogammaglobulinemia is common in patients receiving monoclonal antibody therapy. • In patients with recurrent infections and documented hypogammaglobulinemia, intravenous immunoglobulin (IVIG) replacement therapy should be considered to bolster their immune defenses.^55^

##### Management Strategies

Even with preventive measures, infections can occur. Early recognition and prompt intervention are key to minimizing complications. The relevant management strategies are summarized in **Table 4**.

**Table 4.** Early interventions for infections

*Management Strategies*	*Approach*
Prompt Assessment	• Patients undergoing quadruplet therapy should be educated to promptly report any signs of infection, such as fever, chills, or respiratory symptoms. • Clinicians should maintain a low threshold for initiating further diagnostic workups, including blood cultures, chest imaging, and evaluation of other potential infection sites, particularly in neutropenic patients.
Early Initiation of Broad-Spectrum Antibiotics	• In cases of suspected bacterial infection, early initiation of broad-spectrum antibiotics is crucial, especially in neutropenic patients who are at risk of rapid progression to severe infection or sepsis. • Antibiotic selection should be guided by local epidemiology and patient-specific factors, such as previous infections and antibiotic use.
IVIG Replacement Therapy	• For patients experiencing recurrent bacterial infections and with documented hypogammaglobulinemia, IVIG replacement therapy may be warranted to reduce infection risk and improve quality of life. • Regular monitoring of IgG levels is advised to determine the need for and the effectiveness of IVIG treatment.
Antifungal Prophylaxis and Treatment	• Although not as commonly required as antiviral and antibacterial prophylaxis, antifungal agents (e.g., fluconazole, posaconazole) may be considered in patients with prolonged neutropenia or those undergoing intensive chemotherapy. • Close monitoring for signs of fungal infections, such as persistent fever unresponsive to antibiotics, is also necessary.

##### Conclusion

By implementing a comprehensive strategy that includes prophylaxis, vaccination, and regular monitoring, clinicians can reduce the risk of infections in patients undergoing quadruplet therapy and ensure timely management when infections do occur. This approach is particularly important in transplantation-ineligible patients, who are often at a higher risk of infection due to age, frailty, and more frequent comorbidities. For transplantation-eligible patients, maintaining a balance between aggressive therapy and vigilant infection control is crucial to maximize the benefits of quadruplet regimens while minimizing potential complications. In summary, clinicians are encouraged to review recent consensus recommendations from the EMN, ESCMID, and IMWG regarding infectious disease monitoring and management for NDMM patients receiving quadruplet therapies.

##### Level of Consensus

85.7% (12) agree; 14.3% (2) neutral.

Total: 14 voters.

### Chapter 4: Second ASCT and Maintenance Treatment

#### What are the appropriate maintenance treatment options compatible with the quadruplet therapy regimen among high-risk multiple myeloma (HRMM) patients?

##### Statement

For HRMM patients receiving anti-CD38-based quadruplet induction therapy combined with tandem ASCT, stem cell collection should ideally be planned before the fourth cycle, with early plerixafor administration for patients with poor stem cell mobilization. Additionally, lenalidomide remains the cornerstone of maintenance therapy following induction and ASCT, and a more aggressive maintenance strategy involving lenalidomide plus anti-CD38 isatuximab or daratumumab may be considered in patients with high-risk cytogenetic abnormalities or those who achieved MRD negativity with their initial quadruplet therapy (B1).

##### Discussion

The role of second ASCT and maintenance therapy remains a critical component of the treatment strategy for NDMM. As quadruplet regimens increasingly become the frontline standard of care, important questions emerge regarding their impact on subsequent ASCT success rates and the optimal strategies for maintenance therapy. Quadruplet regimens, combining monoclonal antibodies such as daratumumab or isatuximab, with proteasome inhibitors, immunomodulatory drugs, and corticosteroids, are designed to achieve deeper responses, especially MRD negativity, than traditional triplet regimens, setting the stage for successful subsequent treatments. This depth of response may improve the success rate of second ASCT, as patients with undetectable disease are less likely to experience rapid relapse, making them ideal candidates for another transplant if needed.^56,57^

The efficacy of quadruplet regimens extends beyond just achieving MRD negativity; it also positively impacts PFS and OS.^58^ Clinical data from the Phase II IFM 2018-04 trial suggest that high-risk patients treated with quadruplet regimens such as D-VRd may obtain an improved MRD negativity rate and retain the capacity for a second ASCT with stem cells successfully collected before the initiation of the fourth treatment cycle.^59^ These results underscore the role of quadruplet regimens in improving outcomes not only during the initial phases of treatment but also preserve the options for those who may require additional ASCT transplantation later in their disease course.

Quadruplet therapies are generally well-tolerated, even in intensive settings, with manageable side effects such as neutropenia and thrombocytopenia. The tolerability of these regimens is a key factor in their ability to set the stage for a successful second ASCT, as patients who tolerate the initial regimen well and have their stem cells collected prior to the fourth treatment cycle are more likely to obtain a successful second transplant with MRD-negative outcomes.

The success of a second ASCT, however, is not solely dependent on the prior induction regimen. Patient selection, remission duration, cytogenetic risk factors, and overall fitness must be carefully considered.^60–62^ However, the substantial benefit of a second transplant after a quadruplet therapy incorporating anti-CD38 antibodies may still be debatable, considering the MRD negativity rate were improved only slightly in the IFM 2018-04 trial.^59,63^

##### Maintenance Treatment Options

Maintenance therapy following induction and ASCT is pivotal. The NCCN Guidelines Version 1.2025 have removed all maintenance therapy options for NDMM patients ineligible for transplant. Instead, a continuous treatment strategy is recommended until disease progression, with adjustments to dosage and treatment intensity as needed based on patient tolerance. For maintenance therapy in NDMM patients who are eligible for transplant, lenalidomide remains the preferred treatment option. Additionally, the NCCN Guidelines Version 1.2025 have reclassified the KR and D-R maintenance regimens from “useful in certain circumstances” to “other recommended regimens.” In patients treated with quadruplet regimens, lenalidomide remains the cornerstone of maintenance therapy, given its well-established efficacy.^64,65^ In the phase III AURIGA trial, anti-CD38 naïve post-transplant patients were randomized to receive either daratumumab plus lenalidomide (D-R) or lenalidomide (R) as maintenance treatment.^66^ In this study, a higher MRD negativity was achieved in the D-R maintenance group, suggesting that incorporating anti-CD38 in the maintenance therapy could be beneficial. The subjects in the AURIGA trial had not been exposed to anti-CD38 antibodies prior to enrollment. Moreover, daratumumab-based maintenance therapy has also been demonstrated with improved PFS and MRD negativity as revealed in the CASSIOPEIA trial part 2.^67^ Maintenance therapy following the second ASCT involving anti-CD38 may be considered to prolong disease control and enhance PFS compared with observation group. Dose adjustments should be made as needed to manage any AEs, ensuring that the patient can maintain the therapy over the long term. The second part of the CASSIOPEIA trial did not include a direct head-to-head comparison between daratumumab and lenalidomide monotherapy as maintenance therapy. Similarly, the phase II GRIFFIN trial reported favorable outcomes with D-L for maintenance therapy, but its findings are complicated by differences in the initial regimens used (quadruplet therapy versus triplet therapy). Consequently, the efficacy of daratumumab or D-R as maintenance therapy following a quadruplet regimen that includes anti-CD38 antibodies remains uncertain and requires further investigation to clarify its role in this context. In the NCCN Guidelines Version 1.2025, other monotherapies and combination therapies for maintenance therapy are also included, including ixazomib (however, with more common Grade 3 AEs compared with placebo), bortezomib plus lenalidomide for high-risk myeloma, and carfilzomib plus lenalidomide for transplantation-eligible patients. Consequently, it should be addressed that for maintenance therapy, lenalidomide is still the backbone for high cytogenetic NDMM. More evidences are still required for other potential maintenance therapies in patients received quadruplet therapy that includes anti-CD38 antibodies.

##### Conclusion

In summary, quadruplet regimens have not only transformed frontline therapy for NDMM but also have the potential to influenced subsequent treatment strategies, including second ASCT and maintenance therapy. Lenalidomide is the cornerstone of maintenance therapy, with anti-CD38-based options showing potential in improving PFS and MRD negativity. Still, uncertain efficacies persist regarding anti-CD38-based maintenance post-quadruplet therapy. A tailored approach that considers patient-specific factors and the depth of response achieved during induction is crucial for optimizing outcomes in this evolving treatment landscape.

##### Level of Consensus on that for HRMM patients receiving anti-CD38-based quadruplet induction therapy combined with tandem ASCT, stem cell collection should ideally be planned before the fourth cycle, with early plerixafor administration for patients with poor mobilization

71.4% (10) agree; 21.4% (3) neutral; 7.1% (1) disagree.

Total: 14 voters.

##### Level of Consensus on that lenalidomide remains the cornerstone of maintenance therapy following induction and ASCT, and a more aggressive maintenance strategy involving lenalidomide plus anti-CD38 may be considered in patients with high-risk cytogenetic abnormalities or those who achieved MRD negativity with their initial quadruplet therapy

85.7% (12) agree; 14.3% (2) neutral.

Total: 14 voters.

##### Discussion

The incorporation of quadruplet therapies, such as those incorporating anti-CD38 monoclonal antibodies (e.g., isatuximab and daratumumab), into the frontline treatment of NDMM has gained consensus in recent years. The addition of anti-CD38 monoclonal antibodies into the traditional VRd-based regimens does not significantly alter the well-established administration schedules or monitoring protocols, as the antibodies are administered alongside existing treatments. Additionally, monoclonal antibodies are generally easy to handle for most oncology teams. For oncologists already familiar with triplet therapies, the transition to quadruplet therapy requires only a minimal learning curve while offering superior outcomes. The key benefit of these four-drug combinations is their ability to significantly increase the rates of MRD negativity, a highly reliable prognostic marker that correlates with improved PFS and OS, especially when compared to traditional VRd-based triplet regimens.

Quadruplet regimens, particularly those evaluated in the IMROZ and PERSEUS trials, have demonstrated their capacity to enhance MRD negativity rates across various patient groups, including those with high-risk cytogenetic profiles. In the IMROZ trial, the addition of isatuximab to the VRd regimen resulted in MRD negativity rates of over 55%, compared to 40.9% with VRd alone.^4^ This improvement translated into a 40% reduction in the risk of disease progression or death, underscoring the importance of achieving deep responses early in treatment. Similarly, the GRIFFIN trial showed that daratumumab combined with VRd increased MRD negativity rates to 64%, further confirming the synergistic effects of monoclonal antibodies when added to a VRd backbone.^5^ Furthermore, the safety of quadruplet regimens is also supported by the extensive clinical trial data. Common AEs, such as IRRs, are typically mild and manageable. Importantly, the addition of anti-CD38 antibodies does not significantly exacerbate the long-term toxicities associated with triplet therapies, such as peripheral neuropathy from bortezomib or cytopenia from lenalidomide. Together with the safety results, these findings establish quadruplet therapies as a more suitable option for long-term disease control, particularly in high-risk patients who have historically experienced suboptimal outcomes with triplet therapies.

In addition to quadruplet therapies, emerging options such as bispecific T cell engagers, chimeric antigen receptor (CAR) T-cell therapies, and antibody-drug conjugates (ADCs), provide promising alternatives for relapsed or refractory multiple myeloma listed in the NCCN Guidelines Version 1.2025. Bispecific T cell engagers, including elranatamab-bcmm and teclistamab-cqyv, showed improved objective response rate (ORR) in relapsed/refractory multiple myeloma patients. CAR T-cells, including idecabtagene vicleucel and ciltacabtagene autoleucel, demonstrated improved tumor responses. Belantamab mafodotin-blmf, a B cell maturation antigen (BCMA)-targeting ADC, shows potential benefits in patients relapse after four prior therapies. These advancements ensure that effective therapeutic strategies remain available even for patients who relapse after intensive frontline therapy.

Beyond their immediate efficacy in improving MRD negativity rates, quadruplet therapies also show promise in optimizing treatment strategies for NDMM patients. One of the key considerations in treatment optimization is the balance between efficacy and tolerability, and the use of MRD negativity as a treatment endpoint offers a potential avenue for this balance. Patients who achieve sustained MRD negativity may benefit from de-escalation strategies, potentially reducing the need for continuous or high-intensity maintenance therapy, thereby minimizing long-term toxicity without compromising outcomes. On the other hand, patients who do not achieve MRD negativity may require treatment intensification or extended use of monoclonal antibody-based maintenance regimens to maintain disease control and improve survival.

The role of MRD negativity as a pivotal treatment endpoint is further emphasized when considering its impact on decisions related to second ASCT. Patients who achieve sustained MRD negativity post-induction may be better candidates for consolidation with a second ASCT, leading to prolonged PFS and OS. This is particularly relevant for high-risk patients, who stand to benefit most from the aggressive disease control offered by quadruplet regimens and subsequent transplants. The ability to maintain MRD negativity post-transplant offers a critical advantage in long-term treatment planning, reducing the likelihood of early relapse, which is a common challenge in high-risk patient populations.

While the clinical benefits of quadruplet therapies are clear, there are also challenges associated with their implementation. The addition of a monoclonal antibody to the VRd regimen increases the cost of treatment, which may pose barriers in certain healthcare settings. Additionally, the increased toxicity associated with four-drug regimens, while generally manageable, requires careful patient selection and close monitoring to mitigate AEs. Future research should focus on identifying biomarkers that can predict which patients are most likely to benefit from quadruplet therapy, allowing for more personalized treatment approaches that maximize efficacy while minimizing unnecessary toxicity.

In summary, the use of quadruplet therapies, particularly those incorporating anti-CD38 monoclonal antibodies such as isatuximab or daratumumab, represents a significant advancement in the treatment of NDMM. These regimens consistently achieve higher rates of MRD negativity. By leveraging MRD status as a critical treatment endpoint, clinicians can develop more individualized treatment strategies that reduce toxicity while improving survival. As ongoing research continues to refine the role of MRD and the optimal use of quadruplet therapies, this consensus will likely play an increasingly central role in the management of NDMM, especially for high-risk patients.

### Authors’ Contribution

Conceptualization: Wenming Chen (Lead). Resources: Wenming Chen (Equal), Zhen Cai (Equal), James CS Chim (Equal), Wee Joo Chng (Equal), Juan Du (Equal), Chengcheng Fu (Equal), Ichiro Hanamura (Equal), Jian Hou (Equal), Jeffrey Shang-Yi Huang (Equal), Tadao Ishida (Equal), Aijun Liu (Equal), Vadim Ptushkin (Equal), Anastasiya Semenova (Equal), Naoki Takezako (Equal), Raymond Siu Ming Wong (Equal). Writing – original draft: Wenming Chen (Lead). Writing – review & editing: Zhen Cai (Equal), James CS Chim (Equal), Wee Joo Chng (Equal), Juan Du (Equal), Chengcheng Fu (Equal), Ichiro Hanamura (Equal), Jian Hou (Equal), Jeffrey Shang-Yi Huang (Equal), Tadao Ishida (Equal), Aijun Liu (Equal), Vadim Ptushkin (Equal), Anastasiya Semenova (Equal), Naoki Takezako (Equal), Raymond Siu Ming Wong (Equal).

### Competing of Interest – COPE

The authors declare that they have no known competing financial interests or personal relationships that could have appeared to influence the work in this paper

### Ethical Conduct Approval – Helsinki – IACUC

(for more information, read https://chi.scholasticahq.com/for-authors)

Not applicable.

### Informed Consent Statement

All authors and institutions have confirmed this manuscript for publication.

### Data Availability Statement

Not applicable.
